# Human PIEZO1 Ion Channel Functions as a Split Protein

**DOI:** 10.1371/journal.pone.0151289

**Published:** 2016-03-10

**Authors:** Chilman Bae, Thomas M. Suchyna, Lynn Ziegler, Frederick Sachs, Philip A. Gottlieb

**Affiliations:** Department of Physiology and Biophysics, 302 Cary Hall, State University of New York at Buffalo, Buffalo, NY, United States of America; Xuzhou Medical College, CHINA

## Abstract

PIEZO1 is a mechanosensitive eukaryotic cation-selective channel that rapidly inactivates in a voltage-dependent manner. We previously showed that a fluorescent protein could be encoded within the hPIEZO1 sequence without loss of function. In this work, we split the channel into two at this site and asked if coexpression would produce a functional channel or whether gating and permeation might be contained in either segment. The split protein was expressed in two segments by a bicistronic plasmid where the first segment spanned residues 1 to 1591, and the second segment spanned 1592 to 2521. When the “split protein” is coexpressed, the parts associate to form a normal channel. We measured the whole-cell, cell-attached and outside-out patch currents in transfected HEK293 cells. Indentation produced whole-cell currents monotonic with the stimulus. Single channel recordings showed voltage-dependent inactivation. The Boltzmann activation curve for outside-out patches had a slope of 8.6/mmHg vs 8.1 for wild type, and a small leftward shift in the midpoint (32 mmHg vs 41 mmHg). The association of the two channel domains was confirmed by FRET measurements of mCherry on the N-terminus and EGFP on the C-terminus. Neither of the individual protein segments produced current when expressed alone.

## Introduction

The Piezo proteins are eukaryotic mechanosensitive cation channels that rapidly inactivate in a voltage dependent manner [1, 2] and are gated by membrane tension [3, 4]. Based on the recent CryoEM structure, the channel is a homotrimer [5], although biochemical data had previously suggested a homotetramer [6]. The C-terminal of the channel is necessary for the formation of the pore [7].

The physiological function of Piezo channels is currently under study. This protein was originally designated *Fam38a* and implicated in integrin activation and cell adhesion [8]. In Drosophila, Piezo1 was necessary for a rollover response of the larvae, and ablation of the gene reduced nociception [9]. Genetic diseases, like Xerocytosis [10, 11] involve mutant Piezo1 channels [12, 13] that affect red cell volume regulation [14]. Piezo1 has a role in fluid shear stress response of the vascular endothelium and is critical for development of the vascular system [15, 16]. Piezo1 is required in the lineage choice of stem cell to neuron/astrocyte differentiation [17] and in sensing nanoroughness of the environment [18]. The related protein, Piezo2, is involved in the touch response of Merkel cells [19–21] and proprioception [22]. Interestingly, the mechanical response of chondrocytes appeared to involve a synergy between Piezo1 and Piezo2 [23].

In this work we show that the PIEZO1 protein can be split in two, such that when coexpressed, form a functional wild type channel. These whole-cell currents were robust and monotonic with the stimulus. In cell-attached mode, we observed voltage-dependent activation, slowing with depolarization. For outside-out patches we calculated the Boltzmann curve of activation and showed that the slope sensitivity was similar to wild type, but had a small leftward shift (to lower pressures) in the gating curve. The proximity of the two protein segments was confirmed by energy transfer (FRET) between two fluorophores.

## Materials and Methods

### Electrophysiology

HEK-293 cells (purchased from ATTC) transfected with 500 ng cDNA using TransIT-293 reagent (Mirus), were tested between 24-48h later. All recordings were done as previously described [2, 12, 24, 25]. The pipette solution contained (in mM) 160 KCl, 0.25 EGTA and 10 HEPES and pH was adjusted to 7.3 with KOH for both cell attached and whole cell experiments. The bath solution for cell attached mode contained (in mM) 165 KCl, 1 MgCl_2_, 1 CaCl_2_, 10 HEPES, pH 7.3 (adjusted with KOH) and 160 NaCl, 5 KCl, 1 MgCl_2_, 1 CaCl_2_, 10 HEPES, pH 7.3 (adjusted with NaOH) for whole cell mode. Mechanical stimulation for patches was applied by suction for the cell-attached configuration and positive pressure for the outside-out configuration using an ALA high speed pressure clamp (HSFC-1, ALA Scientific instruments) controlled by QuBIO software (www.qub.buffalo.edu). Whole-cell mechanical stimulation utilized a fire-polished glass pipette (diameter of 2–4 μm) positioned at an angle of 30° with respect to the cover glass. The probe was coarsely positioned ~20 μm from the cell using an MP-285 manipulator (Sutter Instruments Co.), and from that position the probe had vertical movements using a trapezoidal waveform with a piezoelectric stage (P-280.20 XYZ NanoPositioner, Physik Instrumente). The indentation depth (with 40 nm resolution) was controlled using a home built LabVIEW program. The probe velocity was 0.15 μm/ms during the transitions, and the stimulus was held constant for 300 ms. Currents were typically recorded at a holding potential of -60 mV at room temperature. The experiments were performed using an Axopatch 200B amplifier (Molecular Devices), sampled at 10 kHz and filtered at 1 kHz. Data acquisition and stimulation were all controlled by QUBIO and analyzed by QuB software.

### PIEZO1 NtCt construct

A Spe1 site was first inserted into pIRES2-EGFP (after the GFP) by site-directed mutagenesis with the following primers:

qcIEGFPSpeF GCATGGACGAGCTGTACAAGACTAGTTAAAGCGGCCGCGACTCTAG

qcIEGFPSpeR CTAGAGTCGCGGCCGCTTTAACTAGTCTTGTACAGCTCGTCCATGC

To incorporate the protein portion from the N terminal to the end of mCherry, we used human [11] PIEZO1-1591-mCherry vector as a template (covalently linked mCherry to the end of the N-terminal). To prevent the reverse primer from also binding to EGFP on this vector, this DNA was removed by treatment with endonucleases, BamH1 and Not1. The desired DNA fragment was purified by agarose gel electrophoresis, and was amplified with the following primers:

InFIENdeFwd–ATCAAGTGTATCATATGCCAAGTAC

InF1591mChBamR–GGAGAGGGGCGGATCCTACTTGTACAGCTCGTCCATGC

PCR was performed using Prime Star GXL DNA polymerase according to manufacturer’s specification. This 5.9kb product was inserted into Spe1 modified pIRES2-EGFP treated with the endonucleases Nde1 and BamH1, by InFusion HD cloning (Clontech/Takara).

The C-terminus of HekP1 was amplified from amino acid 1591 to the end using Prime Star GXL DNA polymerase and the following primers:

InFHP1ctSpeF–GCTGTACAAGACTAGTAGTGGGCTGGGCGCGGAGGAG

InFHP1ctSpeR–GGCCGCTTTAACTAGTCTACTCCTTCTCACGAGTCCACT

The 2.8kb product was inserted into the vector containing the N-terminal fragment at the Spe1 site by InFusion HD cloning.

### hPIEZO1 FRET construct

To prevent priming from a secondary site, the DNA encoding GFP was removed from *Nt1591mCh IE GFP* vector with the endonucleases BamH1 and Spe1. This DNA was then used as template for PCR to amplify a segment from Nde1 site in the CMV promoter to Age1 site after mCherry (contains DNA that codes for PIEZO1 protein 1–1591 linked to mCherry). A plasmid DNA with GFP inserted into position 1591 was digested with the endonucleases Nde1 and Age1. The PCR produce (1–1591 linked to mCherry) was combined with the plasmid (Nde1/Age1) and ligated by InFusion HD.

### NtCt double mutant construct

The IRES HP1 double mutant C-terminus [24] was amplified using forward and reverse primers having Spe restriction sites and gel purified. The plasmid DNA of the N-terminal of PIEZO1 linked to mCherry was linearized with the endonuclease Spe1. The plasmid and the PCR product were ligated by InFusion HD.

### FRET measurements

Cells were visualized on a Zeiss Axio Observer Z1 inverted microscope with a 63x 1.4 NA oil immersion objective, DIC optics, motorized Z-axis and DefiniteFocus Z-axis positioning (Oberkochen, Germany). Cells expressing the GFP and mCherry fusion proteins were excited with the BDX (450-490nm) and BGX (505-545nm) LEDs (respectively) from an X-Cite XLED1 illuminator, Excelitas Technologies (Waltham, MS). The filter sets in the Zeiss microscope contained: for GFP—a 480/20x excitation filter and a 495lp dichroic, and for mCherry—a 560/55x excitation filter, a 595lp dichroic and a 645/75m emission filter. Images were collected with an Andor iXon DV897U cooled, EM multiplying, back illuminated, CCD camera (Andor, South Windsor, CT). A Dual-View splitter was mounted in front of the camera for simultaneous imaging of GFP and mCherry fluorophores. The Dual-View contained a 565lpxr splitter with 525/50 (GFP) and 680/35 nm (mCherry) emission filters. Images were acquired at 525 nm and 680 nm for both 480 nm and 560 nm excitation wavelengths for a total of 4 images of each cell. FRET ratio was the slope of the 680 nm_(ex480)_/680 nm_(ex560)_ images. The ratio of the 520 nm _(ex480)_ to 680 nm_(ex560)_ was used to confirm that the ratio of expression levels of the two fusion proteins in different cells remained constant. Image acquisition and microscope components were controlled using MicroManager software [26], and image analysis was performed with ImageJ software (rsb.info.nih.gov/ij/).

## Results

**Fig 1:**
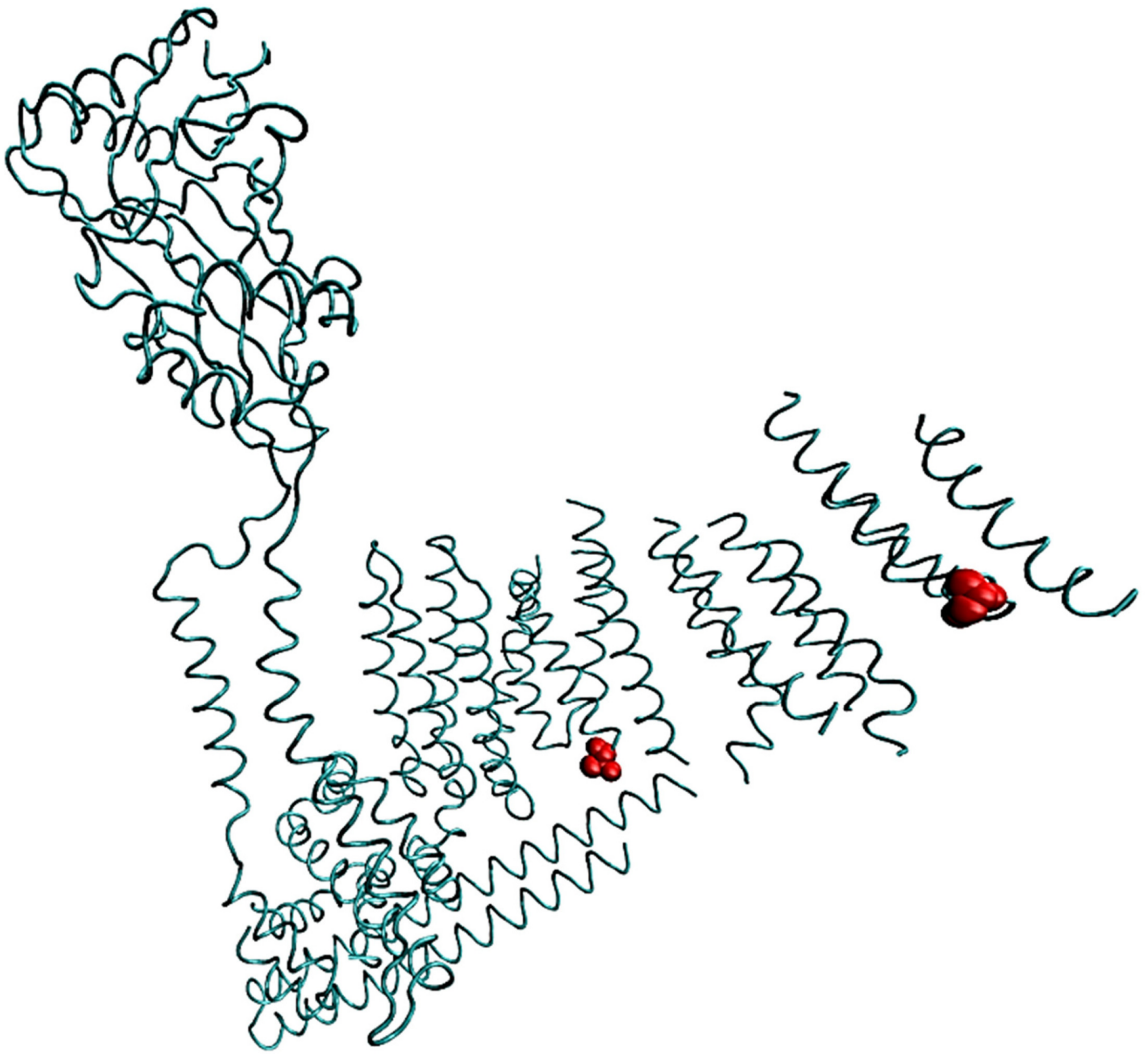
Approximate site where PIEZO1 is split into two protein domains based on the cryoEM structure of mouse Piezo1. The amino acid 1591 is in a region that is disordered. Residues in red are positions 1514 and 1739 (mouse numbering) that border the disordered region and indicate approximately where the fluorescent protein resides. One subunit of the trimer is shown.

We have previously shown that a fluorescent protein could be encoded in the hPIEZO1 protein without disrupting the properties of the channel [3]. In this work we divided the protein into two segments and asked whether the channel function might be reconstituted. The protein was split at position 1591 using a single vector that allowed bicistronic expression of both segments (1 to 1591 and 1592 to 2251 –Fig 1). The production of the N terminus segment (1–1591) was from the first translational start site with an in-frame protein mCherry covalently linked at the end. The synthesis of the C terminus (1591–2251) protein segment was driven by the IE element, and we encoded GFP linked to position 1592. We refer to this vector as hPIEZO1 NtCt. Transfection of cells with this plasmid produced cells that were fluorescent in both red and green, indicating the presence of both segments. We tested these cells for activity with whole-cell, cell-attached and outside-out patches.

**Fig 2:**
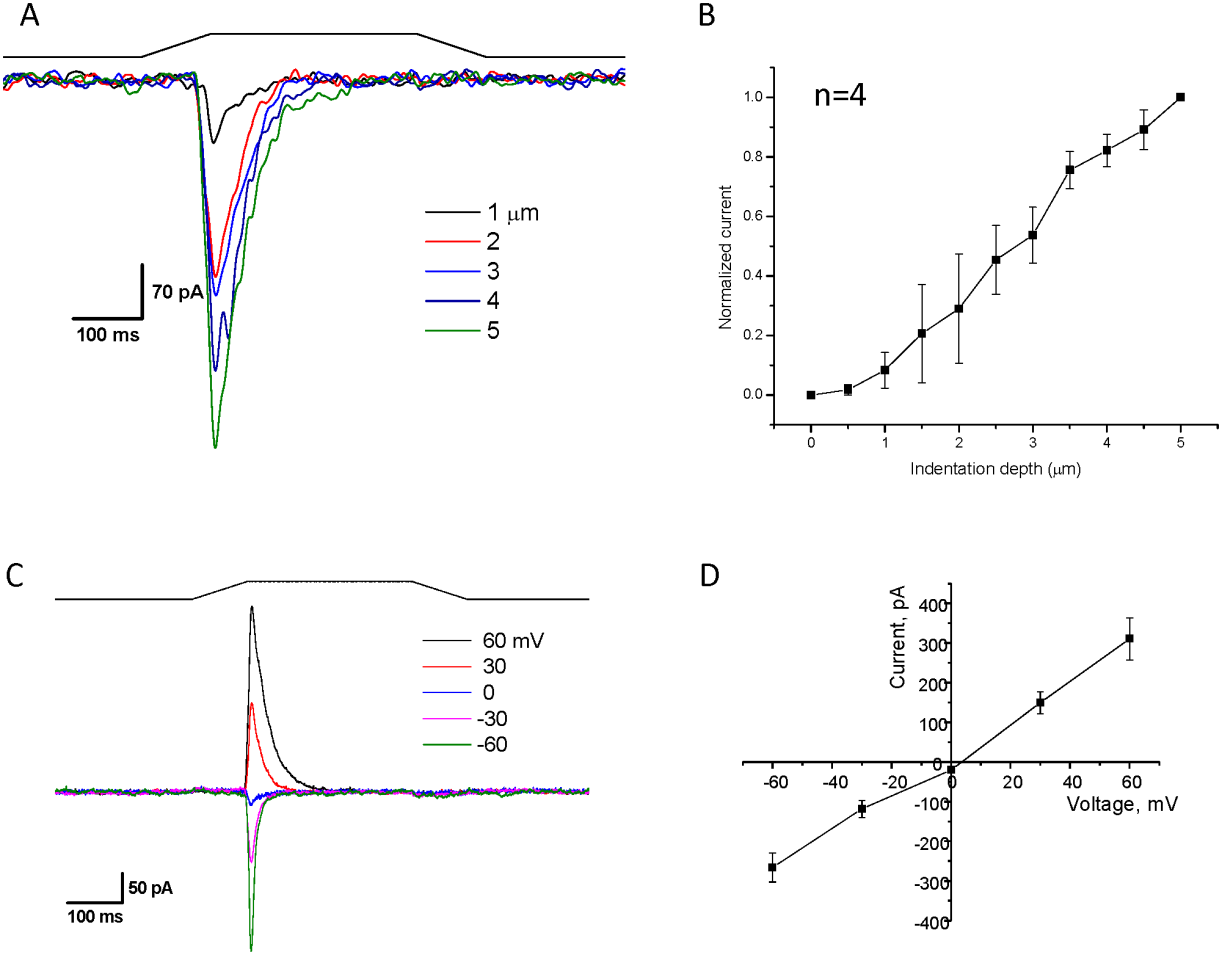
Two hPIEZO1 segments reassemble into a functional channel. A plasmid allowing for the expression of two protein segments (1–1591 with a mCherry protein attached to its C-terminal and 1592–2521 with an N-terminal GFP) was transfected into HEK293T cells. Cells were identified by both green and red fluorescent indicating that both parts of the protein were expressed. **Panel A** shows whole cell currents elicited with a fire-polished probe that mechanically stimulates the cell by pressing on the membrane (holding potential = -60 mV). The black line indicates the stimulus pulse. **Panel B** demonstrates that increasing depth of penetration produces more current (n = 6, error bars are S.D.) **Panel C** is a cell-attached current trace at the indicated voltages, and **Panel D** is a plot of the current versus voltage showing that the channel reverses around 0mV and is similar to the wild type channel (n = 4. error bars are S.D).

We transfected HEK293 cells with 250–500 ng of plasmid (hPIEZO1 NtCt), and after 24–48 hrs measured whole-cell currents at -60 mV (Fig 2) using a fire-polished glass pipette to press on the cell (Fig 2A). A dose-response plot of the current as a function of probe depth is shown in Fig 2B. Fig 2C shows the whole-cell current as a function of membrane potential with a constant mechanical stimulus. A plot of the peak currents from Fig 2C shows that the reversal potential was near 0 mV (Fig 2D), consistent with the wild-type response.

**Fig 3:**
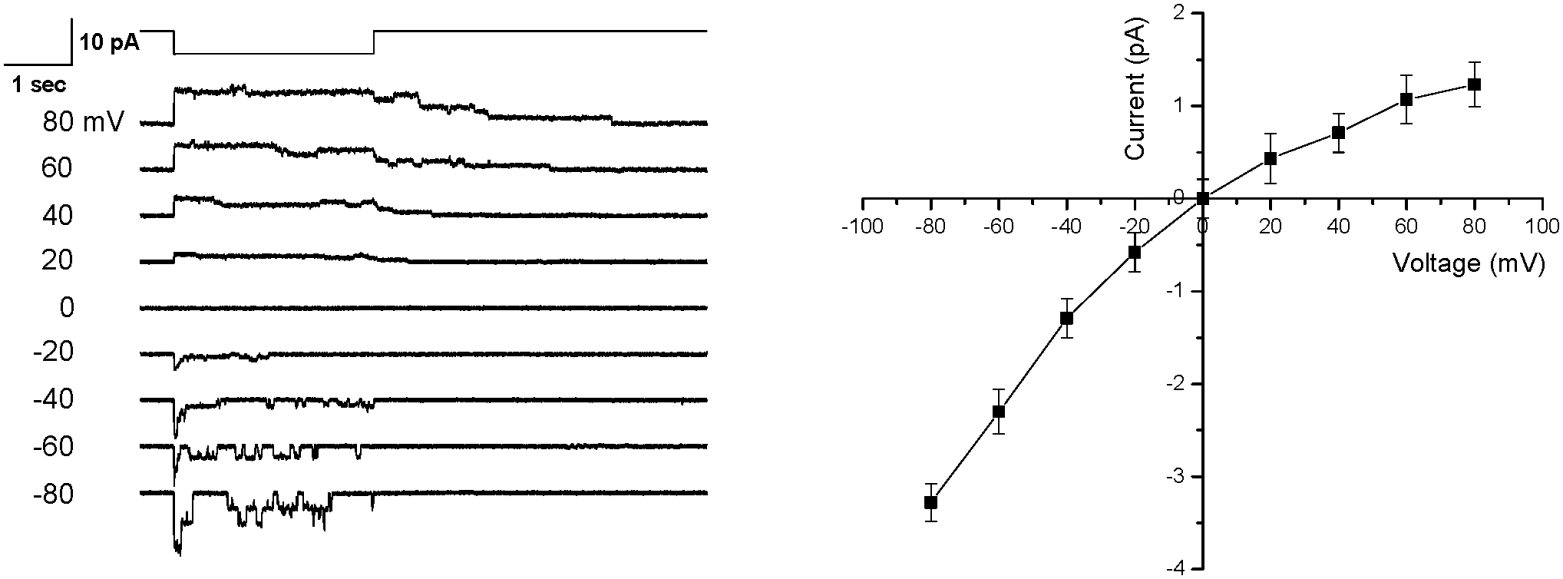
Voltage-dependent inactivation of the hPIEZO1 split protein. Single channel recording of the split protein was measured between 80 to -80 mV membrane potential (**left panel**). For negative membrane potentials we observed rapid inactivation. The mechanical stimulus was suction at -30 mmHg as shown above the current traces. A plot of single channel currents as a function of voltage (**right panel**) showed a reversal potential at ~ 0 mV. (n = 4, error bars are the S.D.).

Results shown in Fig 3 demonstrate that the inactivation rate of the split protein is voltage- dependent (Left-compare currents at -80 and + 80 mV) and is similar to wild-type [1]. Fig 3 is a plot of the single channel current as a function of voltage (Right) and confirms that the reversal potential is near 0 mV. The K^+^ conductance in the absence of divalent cations was 49 pS at -100mV, similar to the wild type channel [27].

**Fig 4:**
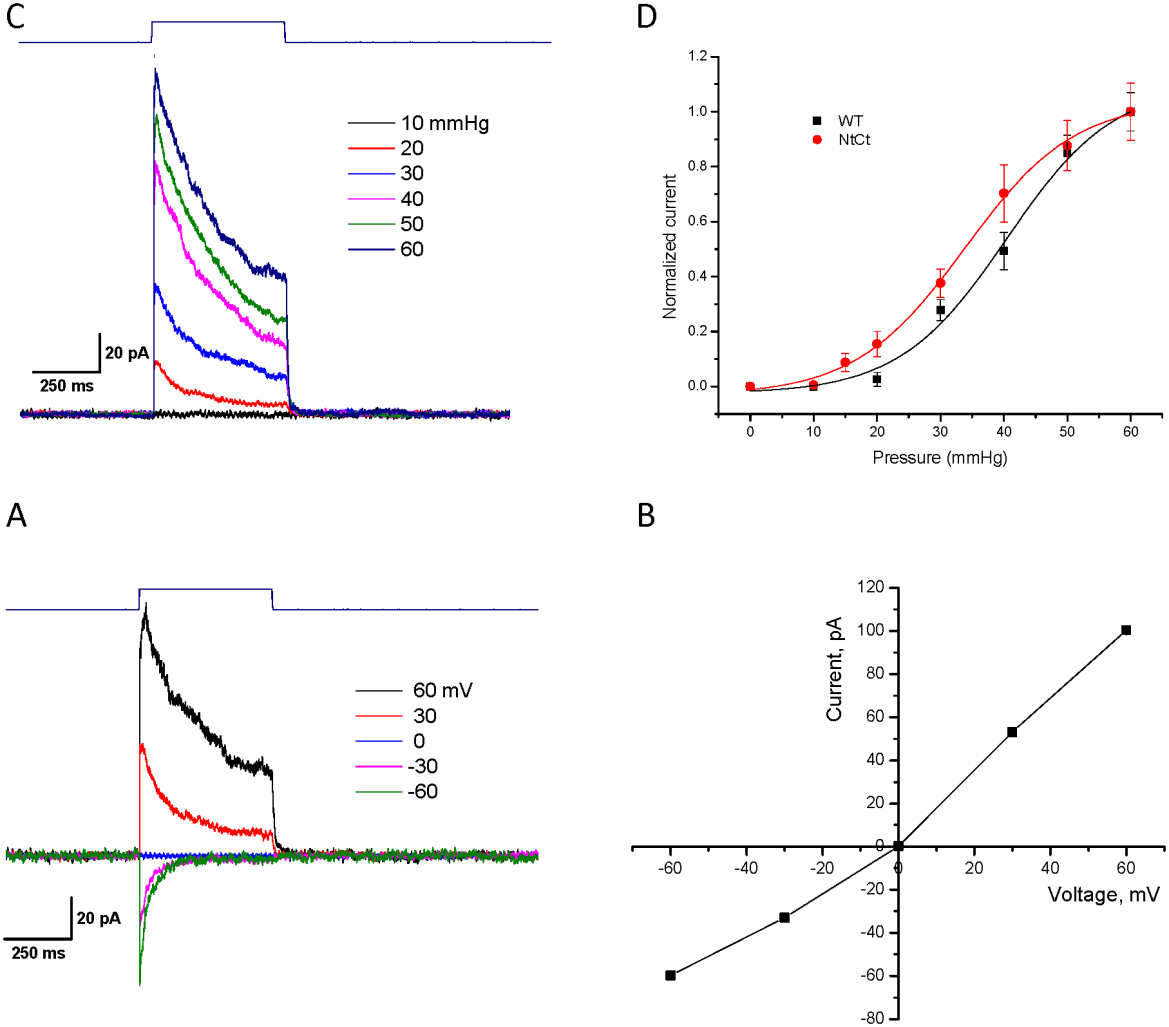
PIEZO1 split protein produces currents from outside-out patches. **Panel A** show that by increasing pressure on outside-out patches is correlated with increasing current. **Panel B** is a Boltzmann plot of current as a function of increasing pressure (n = 6, error bars are the S.D). The slope was approximately 8.6 mmHg^-1^ and P_1/2_ = 34 mmHg, close to wild type values of 8.1 mmHg^-1^ and 42 mmHg, respectively. **Panel C** is the current at the indicated voltages and **Panel D** is a plot of the IV curve showing that the reversal potential for the outside-out patch was ~ 0 mV.

We also analyzed outside-out patches. Fig 4A showed the current increasing with positive pressure pulses. The mean peak current is plotted as a function of the pressure (Fig 4B), and we determined the half-maximal pressure for activation (P_1/2 ‾_ Boltzmann) to be 34 ± 0.9 mmHg (R^2^ = 0.99), slightly leftward shifted compared to wt at 40 ± 2.1 mmHg (R^2^ = 0.99) [12]. There was *no* significant change of the slope (sensitivity) which was ~ 8.5 ± 1.8 vs 8.6 ± 0.9 mmHg^-1^ for wt (Fig 4B) indicating that the conformational change between closed and open states was the same for the wt and split protein. The reversal potential of the split protein channels was near zero (Fig 4C and 4D). Neither the N-terminal nor the C-terminal fragment, when expressed independently, produced visible currents.

**Fig 5:**
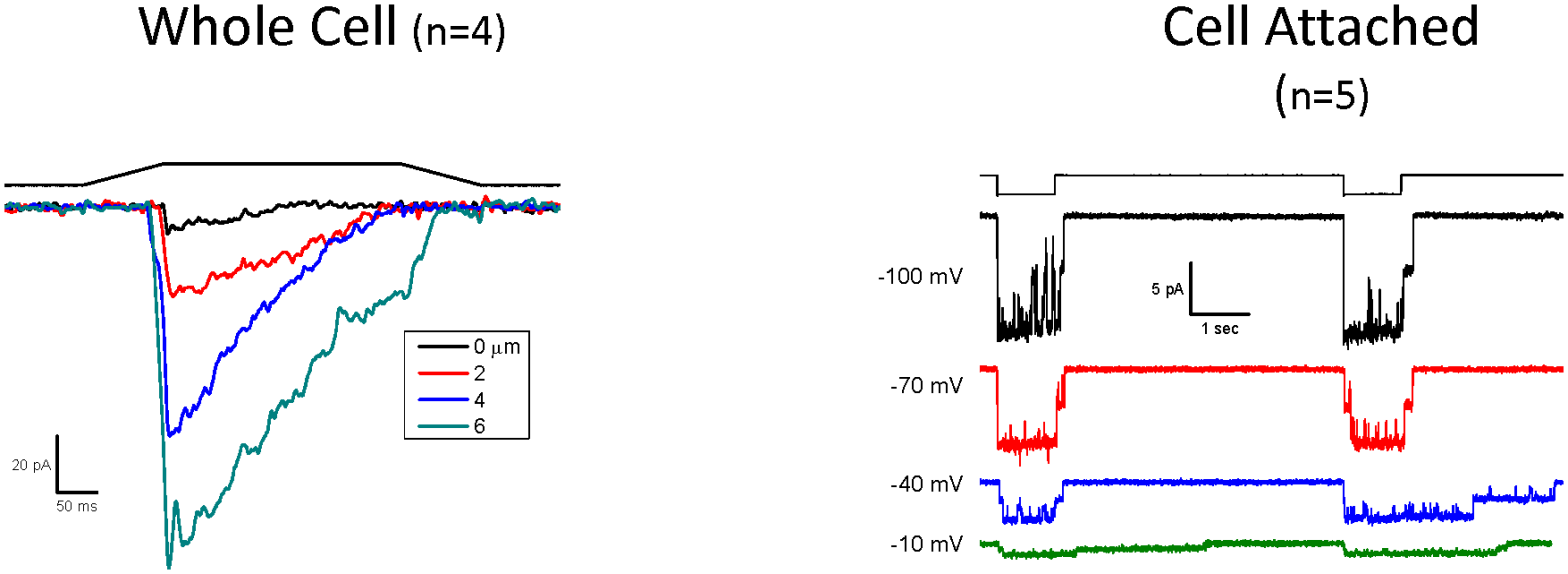
Mutations to the C-terminal fragment alter the inactivation properties of the split protein channel. Two mutations were made to the C-terminal fragment called M2556R and R2456K that are known to remove inactivation in the intact protein. When we expressed the split protein with the mutations made to the C-terminus, we observed an apparent slowing of inactivation for whole-cell (left) and for the single-channel currents (right) there was a loss of inactivation. Whole-cell recording was done at -60 mV with the indicated stimulus pulse above the current traces. Single-channel recordings were done at the indicated voltages with a suction of -30 mmHg.

We have previously shown that two mutations made to the C-terminus remove inactivation[24]. When we introduced these two mutations, M2225R and R2456K, into the C-terminal fragment of the split protein we observed a loss of inactivation (Fig 5) for both whole cell (Left) and cell-attached currents (Right).

**Fig 6:**
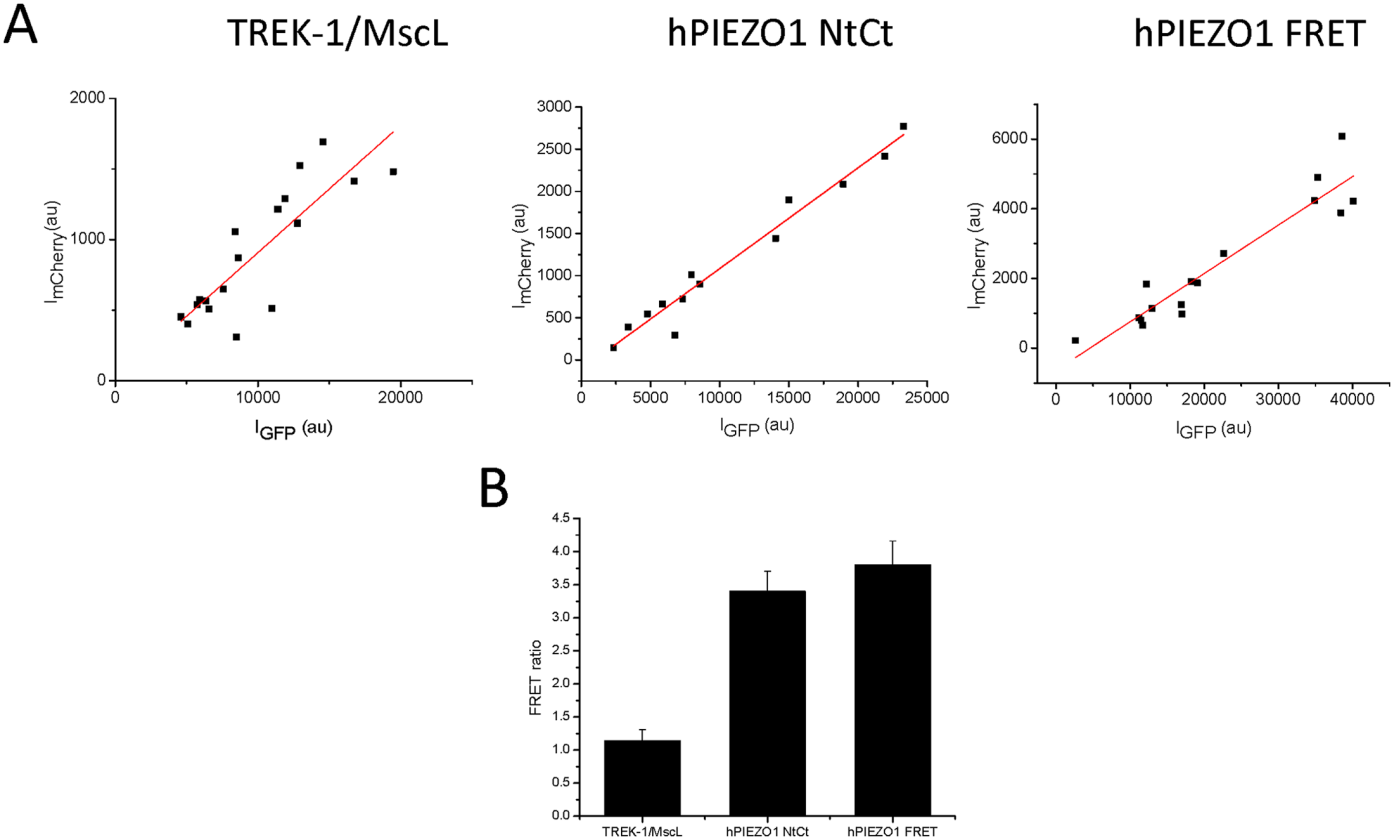
Energy transfer between the two protein segments demonstrates that they are in close proximity. The two fluorophores used to tag the protein segments have spectral overlap,allowing us to measure their proximity by measuring energy transfer (FRET). We transfected HEK293 cells with bicistronic plasmid TREK1-mCherry/MscL-EGFP and hPIEZO1 Nt-mCherry/Ct-EGFP and the hPIEZO1-FRET fusion protein. We measured the emissions of EGFP (525nm) and mCherry (680nm) for multiple cells from each transfection and plotted them in **Panel A**. The data from each transfection were fit by linear regression showing slopes of TREK1/MscL –0.09, hPIEZO1 NtCt– 0.13, and hPIEZO1 FRET– 0.12. The amount of energy transfer between the two fluorophores was determined by the ratio of the 680nm emission excited at 470nm and 560nm (**Panel B**). The ratio of TREK-1/MscL proteins was 1.15 ± 0.17 (n = 18, error bars are S.D.). The ratio for the linked fluorophores in cells expressing hPIEZO1 FRET fusion protein had a value of 3.8 ± 0.37 (n = 16, error bars are S.D.). The ratio for the split protein monomers was 3.4 ± 0.3 (n = 14, error bars are S.D.), which is close to the hPIEZO1 FRET ratio, suggesting the monomers are in close proximity.

To confirm intermolecular interactions between the coexpressed Nt and Ct halves, we determined the proximity of the two proteins by calculating the FRET ratio using the 680 nm emission of mCherry at 480 and 560 nm excitation. As a control for minimal FRET ratio (i.e. minimal energy transfer), we coexpressed TREK1-mCherry and MscL-EGFP, two proteins that have different distributions and are assumed not to interact (S1 Fig). To assess the maximal expected energy transfer, we constructed a second vector that covalently linked the two fluorophores making a contiguous protein called hPIEZO1 FRET. TREK1/MscL and the Nt/Ct halves were transfected on bicistronic pIRIS vectors as described above, allowing coexpression of the pairs of proteins at a stoichiometric ratio even though the overall expression level varied from cell to cell (Fig 6A). The slopes of the intensities of EGFP:mCherry for TREK1/MscL and Nt/Ct from bicistronic expression were similar to the slope for hPIEZO1 FRET expressing cells which have a 1:1 ratio of GFP:mCherry, showing that bicistronic expression produced nearly equal amounts of both proteins.

The ratio of the expected minimal energy transfer in this system was 1.15 ± 0.17 based on the TREK-1/MscL pair (Fig 6B), while the ratio for hPIEZO1 (maximal energy transfer) was 3.8 ± 0.37. The energy transfer for the coexpressed hPIEZO1 Nt and Ct halves was 3.4 ± 0.3 (Fig 6B **NtCt**) supporting nm-scale interactions between the two proteins. The FRET ratio data was supported by the Ct and Nt expression patterns which showed very different distributions when expressed separately (S2 Fig **(Ct) and** S3 Fig **(Nt)**). The Ct half primarily resides in cytoplasmic and nuclear regions, while the Nt half localizes preferentially to membranes. Coexpression of the two halves caused the Ct protein to distribute in similar pattern with the Nt protein, suggesting the Nt protein escorts the Ct protein to more membrane localized regions when the two interact.

## Discussion

We previously showed that encoding a fluorescent protein at position 1591 of hPIEZO1 did not alter the biophysical properties of the channel [3]. The ability to insert a small protein without loss of channel function suggested that PIEZO1 may have at least two independent domains, and that the fluorescent protein sits at the border between them. To test this, we constructed a vector that could synthesize the two domains independently and asked if they could assemble into an active channel. In fact, they did. All of the assays we used showed that the split protein was nearly identical to the wild-type channel. The proximity of the two components in the assembled channel was verified by observing a high FRET efficiency, and efficiency that did not occur with the controls made of TREK-1-EGFP and MscL-mCherry.

The structure of mouse Piezo1 elucidated by cryo EM to 4.8 Å resolution showed that the pore is defined by a trimer of the C-terminal segment [5]. These results are consistent with a mutation in the C-terminal that alters the conductance of the channel [7]. Structural components called “blades” on the extracellular side are part of the N-terminal segment and “beams” reside in the intracellular side. Based on homology between the human and mouse protein, the site at which the protein is split is a disordered intracellular domain [5]. This region is positioned between the C-terminal and the N-terminal domain near the region called the anchor, and because it can accommodate insertion of large proteins without loss of function, it suggests that this region is not critical for the mechanical response of the channel.

The split-protein technique has been applied to the potassium channel, TOK1, where the pore was split in two [28] but the separate domains, which were able to conduct ions, did not assemble into a functional potassium channel. The prokaryotic channel MscL from *Mycobacterium tuberculosis* was also divided into two parts [29], generating a N-terminal half containing TM1 and a C-terminal half containing TM2. Both fragments were tested and only TM1 formed channels that were not sensitive to mechanical stimulation and produced a variety of conductances that were not wild type. When the MscL channel was assembled from both fragments, wild type conductance was restored but the channel was responsive at lower pressures. This is similar to what we observe for the PIEZO1 split protein, where the half-maximal pressure for activation is shifted leftward on the Boltzmann curve. This observation does not necessarily indicate a common mechanism because the work on MscL involves two transmembrane domains that are linked by a periplasmic segment needed for MscL function, while the site of PIEZO1 is distal to the putative pore which is located near the C-terminal.

We were unable to generate channel activity from either PIEZO1 segment when expressed alone. While the Nt segment expressed alone was targeted to membrane domains, the Ct half did not, but became membrane localized with coexpression (see S2, S3 and S4 Figs). This suggests that the Nt segment associates with the Ct segment and escorts it to the plasma membrane. Our results are consistent with the published results that the C-terminus houses the pore [5, 7] and the N-terminus contains the sensor for mechanical gating [5] and the plasma membrane targeting sequences.

## Supporting Information

S1 Fig TREK1 and MscL proteins do not colocalize. Optical sections (0.25 μm thick) showing DIC and fluorescent images of a representative HEK cell expressing TREK1-mCherry and MscL-EGFP bicistronic vector. A section near the glass coverslip and in the middle of the cell are shown. EGFP images at 525 nm (green) and mCherry image at 680 nm (orange). TREK1 primarily labels plasma membrane and ER regions. MscL primarily labels internal membrane structrues like nuclear membrane, vesicles, ER and reticulate stuctures farther from the nucleus. An “*” designates nucleus position, and “#” designates likely endoplasmic reticulum (ER) region.
(TIFF)

S2 Fig Ct-EGFP protein expressed alone shows diffuse, homogenous, labeling of cytoplasmic and nuclear regions with no indication of plasma membrane labeling. Middle section images taken from two groups of cells. (A) shows two cells expressing higher concentration of Ct-EGFP with cytoplasmic and nuclear distribution. (B) Shows two cells with lower expression having primarily diffuse cytoplasmic distribution. An “*” designates nucleus position, and “#” designates likely endoplasmic reticulum (ER) region.
(TIFF)

S3 Fig Nt-mCherry protein expressed alone labels internal and surface membranes, but does not enter the nucleus. Two representative cells are shown (A and B) with image sections near the glass coverslip and in the middle of the cell. Many punctae form on the surface and dense staining occurs in the ER and at the ends of cell protrusions. *In addition*, *most Nt-mCherry expressing cells are highly vesiculated*, *though the reason for this was not investigated further*. An “*” designates nucleus position, and “#” designates likely endoplasmic reticulum (ER) region.
(TIFF)

S4 Fig Ct-EGFP and Nt-mCherry coexpression causes Ct proteins to relocate to Nt regions. While not complete colocalization, most of the Ct protein is localized to Nt regions, and no Ct protein no longer goes to the nucleus. Single representative cell showing image sections near glass coverslip and near the middle of the cell. Cells expressing both halves do not show vesiculation. An “*” designates nucleus position, and “#” designates likely endoplasmic reticulum (ER) region.
(TIFF)
