# Supplementary figures and images for: Human PIEZO1 Ion Channel Functions as a Split Protein
Source: PLoS One. 2016 Mar 10;11(3):e0151289. doi: 10.1371/journal.pone.0151289 (PMC4786117; doi:10.1371/journal.pone.0151289)

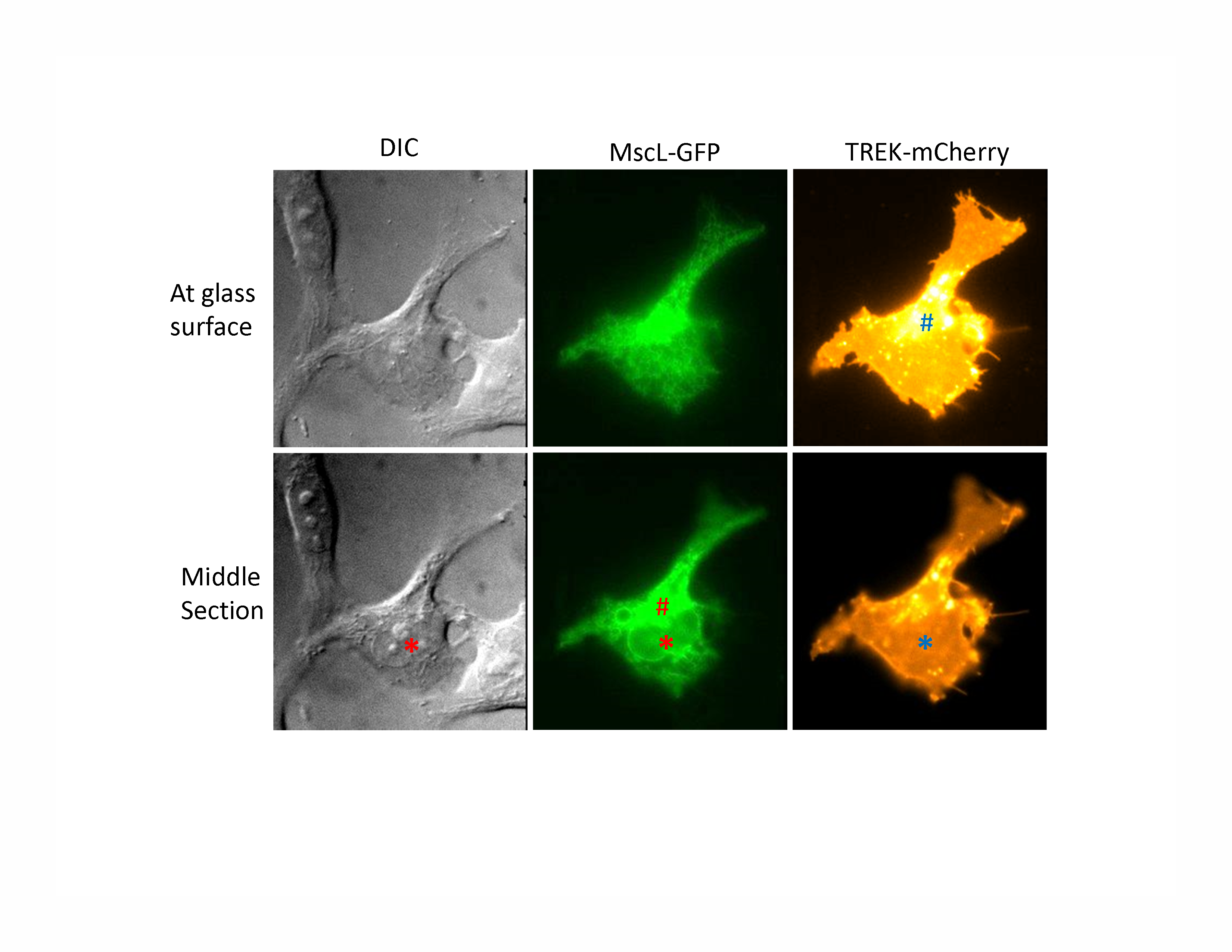

Supplement: S1 Fig — Optical sections (0.25 μm thick) showing DIC and fluorescent images of a representative HEK cell expressing TREK1-mCherry and MscL-EGFP bicistronic vector. A section near the glass coverslip and in the middle of the cell are shown. EGFP images at 525 nm (green) and mCherry image at 680 nm (orange). TREK1 primarily labels plasma membrane and ER regions. MscL primarily labels internal membrane structrues like nuclear membrane, vesicles, ER and reticulate stuctures farther from the nucleus. An “*” designates nucleus position, and “#” designates likely endoplasmic reticulum (ER) region. (TIFF) [file pone.0151289.s001.tiff]

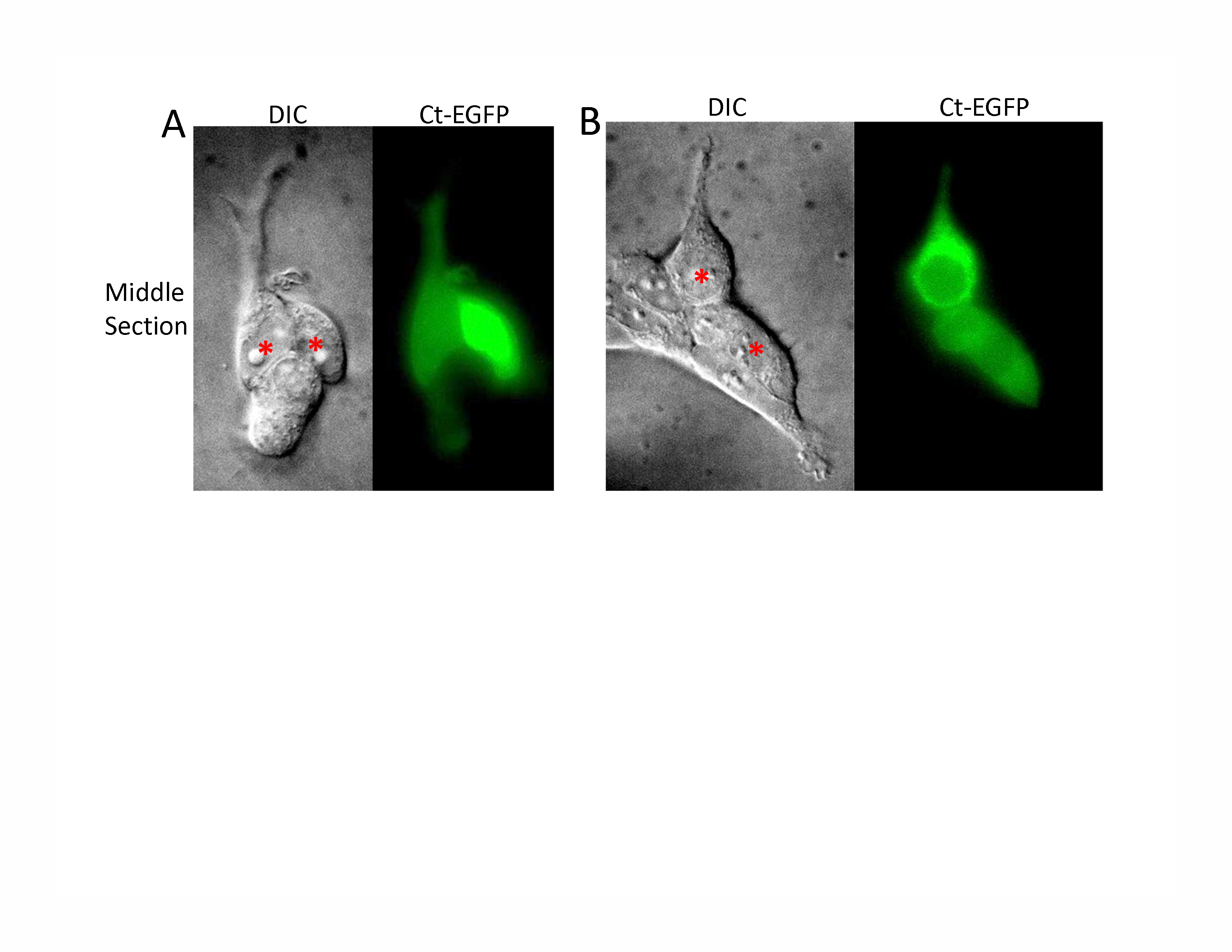

Supplement: S2 Fig — Middle section images taken from two groups of cells. (A) shows two cells expressing higher concentration of Ct-EGFP with cytoplasmic and nuclear distribution. (B) Shows two cells with lower expression having primarily diffuse cytoplasmic distribution. An “*” designates nucleus position, and “#” designates likely endoplasmic reticulum (ER) region. (TIFF) [file pone.0151289.s002.tiff]

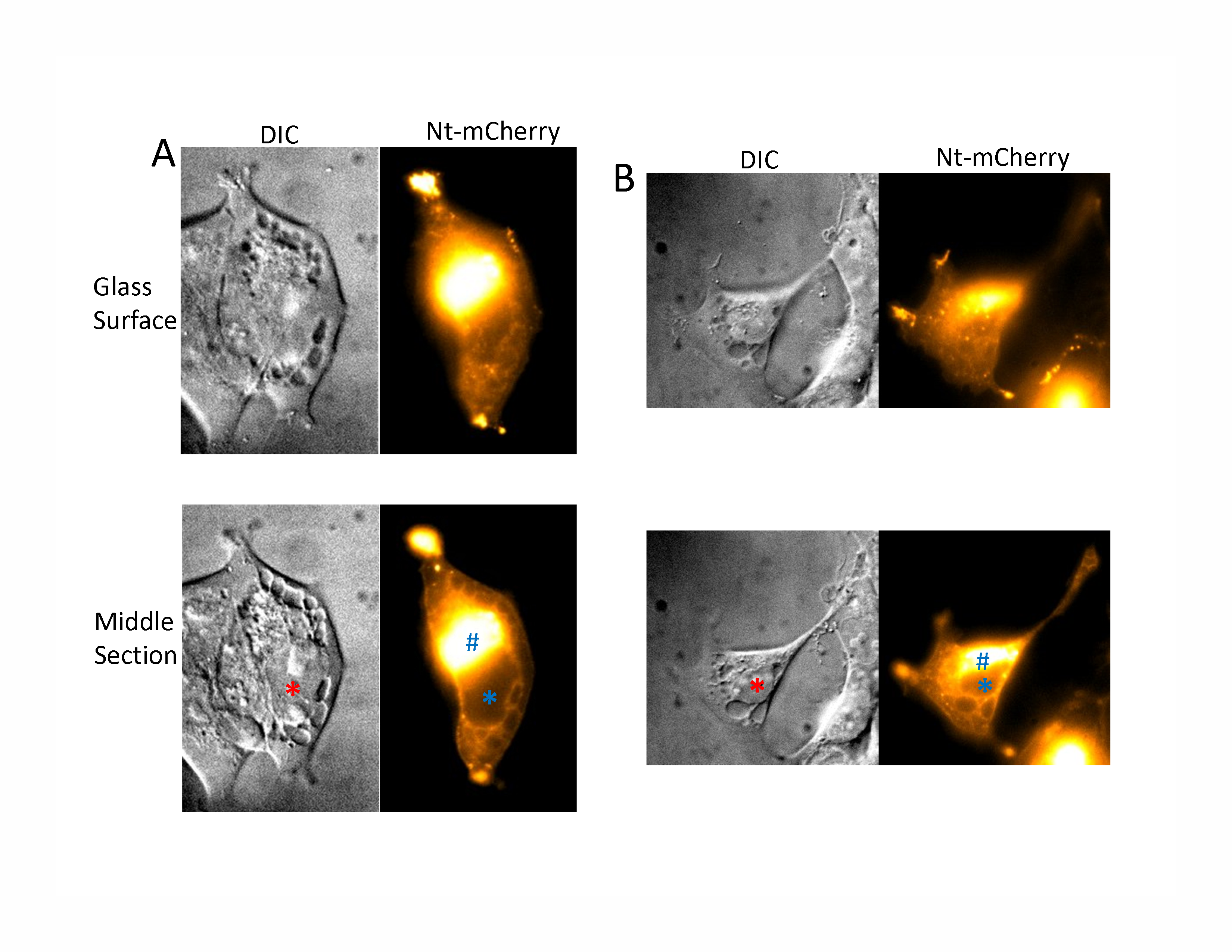

Supplement: S3 Fig — Two representative cells are shown (A and B) with image sections near the glass coverslip and in the middle of the cell. Many punctae form on the surface and dense staining occurs in the ER and at the ends of cell protrusions. In addition, most Nt-mCherry expressing cells are highly vesiculated, though the reason for this was not investigated further. An “*” designates nucleus position, and “#” designates likely endoplasmic reticulum (ER) region. (TIFF) [file pone.0151289.s003.tiff]

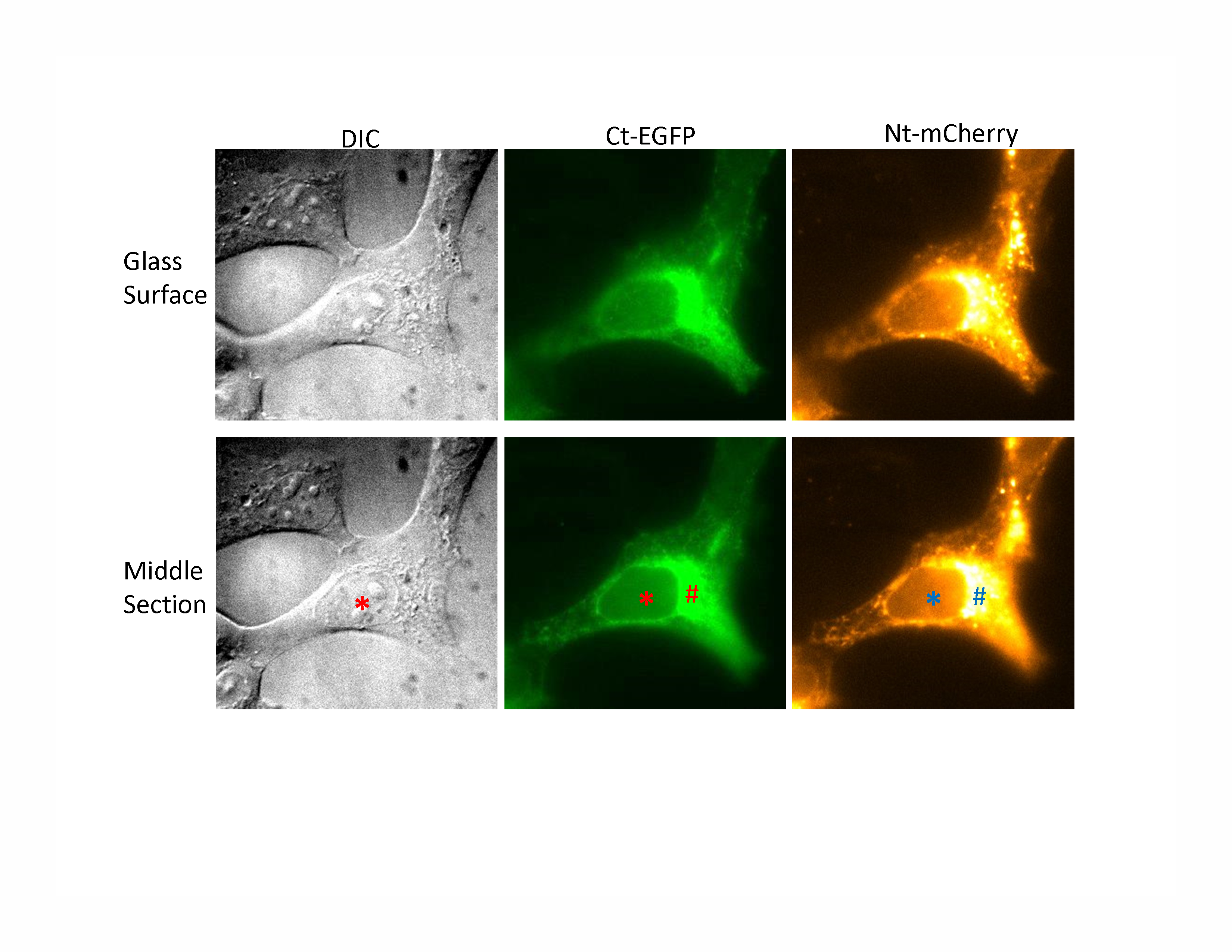

Supplement: S4 Fig — While not complete colocalization, most of the Ct protein is localized to Nt regions, and no Ct protein no longer goes to the nucleus. Single representative cell showing image sections near glass coverslip and near the middle of the cell. Cells expressing both halves do not show vesiculation. An “*” designates nucleus position, and “#” designates likely endoplasmic reticulum (ER) region. (TIFF) [file pone.0151289.s004.tiff]
